# Stroke Incidence in Victoria, Australia—Emerging Improvements

**DOI:** 10.3389/fneur.2017.00180

**Published:** 2017-05-04

**Authors:** Benjamin B. Clissold, Vijaya Sundararajan, Peter Cameron, John McNeil

**Affiliations:** ^1^Stroke Unit, Monash Medical Centre, Clayton, VIC, Australia; ^2^Stroke and Ageing Research Group, Department of Medicine, Monash University, Clayton, VIC, Australia; ^3^Department of Epidemiology and Preventive Medicine, Monash University, Clayton, VIC, Australia; ^4^St Vincent’s Hospital, University of Melbourne, Parkville, VIC, Australia

**Keywords:** stroke, epidemiology, administrative data, cohort, registry

## Abstract

**Background:**

Evidence of a decline in the incidence of stroke has emerged from population-based studies. These have included retrospective and prospective cohorts. However, in Australia and other countries, government bodies and stroke foundations predict a rise in the prevalence of stroke that is anticipated to increase the burden of stroke across the entire domain of care. This increase in prevalence must be viewed as different from the decline in incidence being observed, a measure of new stroke cases. In Victoria, all public emergency department visits and public and private hospital admissions are reported to the Department of Health and Human Services and include demographic, diagnostic, and procedural/treatment information.

**Methods:**

We obtained data from financial years 1997/1998 to 2007/2008 inclusive, for all cases with a primary stroke diagnosis (ICD-10-AM categories) with associated data fields. Incident cases were established by using a 5-year clearance period.

**Results:**

From 2003/2004 to 2007/2008 inclusive, there were 53,425 patients with a primary stroke or TIA diagnosis. The crude incident stroke rate for first ever stroke was 211 per 100,000 per year (95% CI 205–217) [females—205 per 100,000 per year (95% CI 196–214) and males—217 per 100,000 per year (95% CI 210–224)]. The overall stroke rates were seen to significantly decline over the period [males (per 100,000 per year) 227 in 2003/2004 to 202 in 2007/2008 (*p* = 0.0157) and females (per 100,000 per year) 214 in 2003/2004 to 188 in 2007/2008 (*p* = 0.0482)]. Ischemic stroke rates also appeared to decline; however, this change was not significant.

**Conclusion:**

These results demonstrate a significant decline in stroke incidence during the study period and may suggest evidence for effectiveness of primary and secondary prevention strategies in cerebrovascular risk factor management.

## Introduction

Evidence of a decline in the incidence of stroke has emerged from a number of population-based studies in the last two decades (1). These have included both retrospective and prospective cohorts from a variety of different patient groups worldwide. Similar decline is evident in incidence of TIA in some populations (2). In Australia and other countries, numerous organizations involved in stroke planning and management, including government bodies and stroke foundations, predict a rise in the prevalence of stroke, based on evidence of an aging population. This is anticipated to increase the burden of stroke across the entire domain of stroke care, from acute management to rehabilitation and long-term care. This increase in stroke prevalence, the proportion of disease in the population, must be viewed as different from the decline in incidence being observed, a measure of new stroke cases. The decline seen in incidence is a compelling marker of increasing use of primary and secondary prevention strategies in the area of cerebrovascular risk factor management, and a sign of the emerging effectiveness of those therapies.

Many of the studies in this area have included prospectively collected data on stroke incidence. Such approaches are methodologically superior, but time and labor intensive. An additional emerging trend is that many patients with stroke are now managed acutely in hospital, in line with efforts to streamline the completion of acute investigation and early institution of secondary prevention. In most cases, hospitals can provide the timeliest performance of CT and carotid ultrasound for TIA and ischemic stroke, as well as initiating early assessment by skilled stroke clinicians. In Australia, data regarding every episode of care for patients admitted to hospital is collected by government health departments, at a state level, in order to assist with financial allocation and service provision. Such administrative datasets are retrospectively compiled, based on mandatory information provided by individual hospitals, using trained coding staff. These data include primary and secondary diagnostic codes, time and date data, discharge destination and demographic data, among others. Diagnostic codes are collected using the International Classification of Diseases (ICD-10-AM in Australia since 1998).

Administrative data have limitations. It lacks disease-specific data fields, stroke-related comorbidities, stroke severity and clinical signs, admission to stroke units, use of thrombolysis and outcomes of such therapy, imaging details, and reliable functional outcome data. However, with large patient numbers available over many years, these datasets represent a readily available source of information for analysis regarding disease incidence as well as disease management and broad outcomes. The increasing move toward collection of data in a national stroke registry will help to obtain vital stroke-specific metrics, which can inform performance and allow benchmarking across institutions.

Previous community stroke studies have evaluated incidence in smaller, defined regions. Our aim was to use hospital collected administrative data for our state’s population, in order to establish potential trends in stroke incidence.

## Materials and Methods

**Figure 1:**
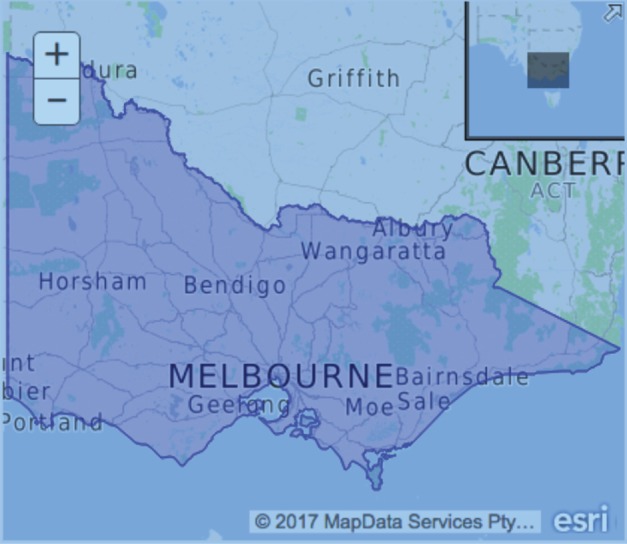
**Victorian map (with thanks, Australian Bureau of Statistics)**.

We obtained data of all hospital admissions from the Victorian Admitted Episodes Dataset (Department of Health and Human Services, Victoria) from financial years 1997/1998 to 2007/2008 inclusive, for the state of Victoria, Australia (Figure 1). The state population, based on Australian Bureau of Statistics census data for 2006, was 4,932,422 (2,420,415 males; 2,512,007 females—includes 1,173,201 born overseas) (3). All cases with a primary stroke or TIA diagnosis (ICD-10 categories) were extracted, with associated data fields. Incident hospital admitted cases were established by using a 5-year clearance period, an assumption that, by capturing only cases without a hospital diagnosis of stroke in the 5 years prior, the remaining cases were true incident cases. Analysis was performed using SAS version 9.2 (Copyright© 2002–2008 by SAS Institute Inc., Cary, NC, USA). This study was approved by the Monash University Human Research Ethics Committee and Department of Human Services (Victoria) Human Research Ethics Committee. Incident rates are expressed as per 100,000 population per year.

## Results

Crude numbers were established for each financial year. Directly standardized numbers were calculated using the age- and sex-matched Australian population numbers for males and females, based on Australian Bureau of Statistics numbers for 2006.

- For the period 2003/2004 to 2007/2008, there were 53,425 patients with a primary stroke or TIA diagnosis.
- 27,132 males and 26,293 females were identified as incident stroke cases, with a primary stroke diagnosis. See Table 1 for patient demographics.
- 42,338 (67.82%) patients with a stroke type diagnosis resided in urban areas versus 18,500 (29.63%) in rural areas.

**Table 1:** **Patient demographics**.

Age	71.7 ± 15.6
Male gender	27,132 (51%)
Atrial fibrillation	5,981 (11%)
Hypertension	22,914 (43%)
Ischemic heart disease	2,496 (5%)
Smoking	6,333 (12%)
Diabetes mellitus	10,360 (19%)

See Table 2 for breakdown of stroke type as per ICD-10-AM classification.

**Table 2:** **Stroke type**.

Primary stroke type (ICD-10)	2003/2004	2004/2005	2005/2006	2006/2007	2007/2008
Cerebral infarction	3,142	3,150	3,396	3,334	3,180
Cerebrovascular disorders in diseases classified elsewhere	5	5	4	3	3
Intracerebral hemorrhage	1,005	928	1,048	950	967
Occlusion and stenosis of cerebral arteries, not resulting in cerebral infarction	60	60	61	58	51
Occlusion and stenosis of precerebral arteries, not resulting in cerebral infarction	927	863	875	811	716
Other cerebrovascular diseases	567	551	589	663	591
Other non-traumatic intracerebral hemorrhage	419	406	408	487	467
Stroke, not specified as hemorrhage or infarction	1,797	1,811	1,681	1,498	1,417
Subarachnoid hemorrhage	418	454	409	447	450
TIA and related syndromes	2,332	2,268	2,471	2,473	2,628
Vascular syndromes of brain in cerebrovascular disease	25	29	19	26	22
Total	10,697	10,525	10,961	10,750	10,492

**Figure 2:**
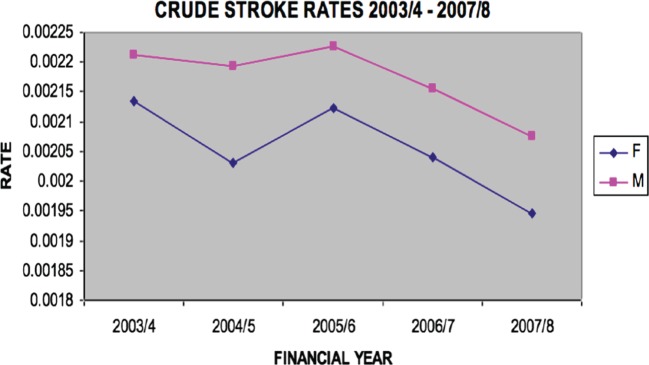
**Crude stroke rate 2003/2004–2007/2008**.

The crude incidence stroke rate for first ever stroke was 211 per 100,000 per year (95% CI 205–217). For females, the rate was 205 per 100,000 per year (95% CI 196–214) and for males the rate was higher, at 217 per 100,000 per year (95% CI 210–224). See Figure 2 for crude stroke rates.

**Figure 3:**
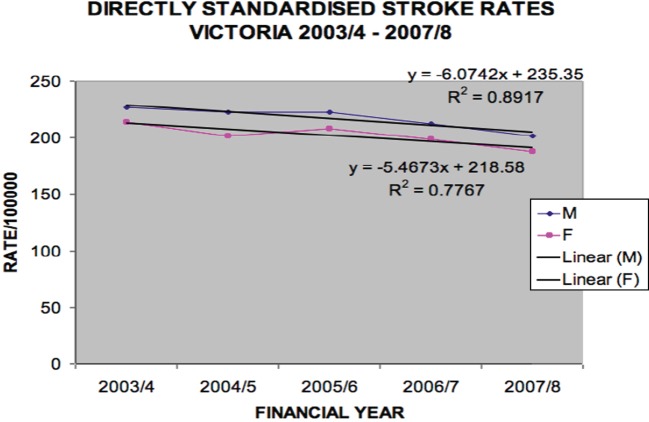
**Directly standardized stroke rates Victoria 2003/2004–2007/2008 (males *p* = 0.0157, females *p* = 0.0482)**.

Data were also analyzed by direct standardization methods for age and sex. Figure 3 shows incident stroke rates following direct standardization.

The overall (ischemic and hemorrhagic) stroke rates declined over the period [males (per 100,000 per year) 227 in 2003/2004 to 202 in 2007/2008, *p* = 0.016 and females (per 100,000 per year) 214 in 2003/2004 to 188 in 2007/2008, *p* = 0.048].

**Figure 4:**
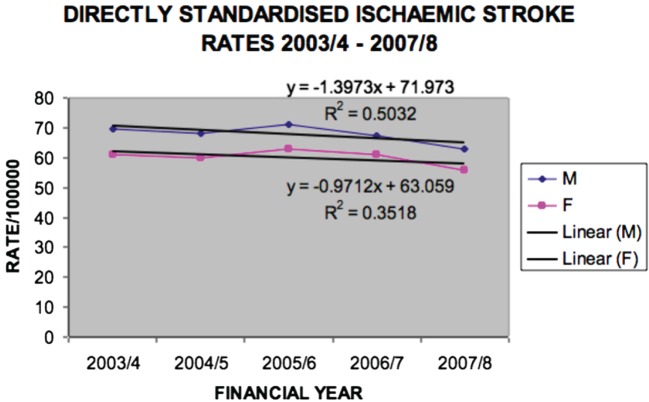
**Directly standardized ischemic stroke rates Victoria 2003/2004–2007/2008 (males *p* = 0.1797, females *p* = 0.2918)**.

**Figure 5:**
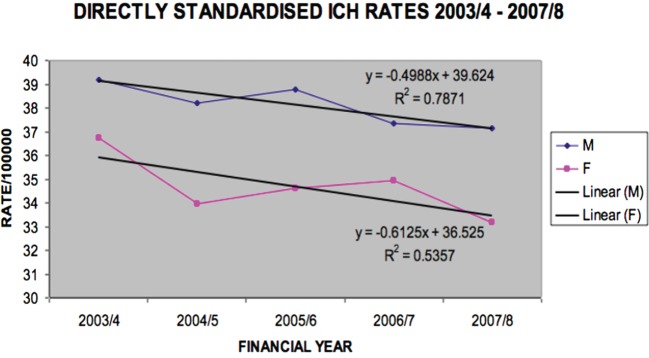
**Directly standardized intracerebral hemorrhage rates Victoria 2003/2004–2007/2008 (males *p* = 0.0447, females *p* = 0.1598)**.

Ischemic stroke rates did not significantly decline (Figure 4), but hemorrhagic stroke rates did for males (*p* = 0.045) (Figure 5).

Overall in-hospital mortality rates for the same period did not change significantly for ischemic (*p* = 0.0665) or hemorrhagic (*p* = 0.4221) stroke.

## Discussion

Population and community data suggest that stroke incidence may be declining in the developed world. Evidence from hospitalization data and community cohort studies confirms this trend (4) (see Table 3). Thrift et al., using hospitalization episode data and age- and gender-specific mortality data, found that despite expected predictions of increasing stroke hospitalization and mortality (based on hospitalization and mortality data from 1996/1997), actual figures from 2005/2006 indicated a 2.21% annual decline in hospitalization rate and 4.0% annual reduction in mortality rate. Data from the Perth Community Stroke Study showed a similar picture of declining incidence. Compared with the 1989–1990 period, gender- and age-adjusted incidence rates declined by 25% (95% CI 10–37%) in 1995–1996 and by 43% (95% CI 31–53%) in 2000–2001, corresponding to a 5.5% average annual decrease overall (1, 5, 6). Stable early case-fatality rates and declining frequency of key vascular risk factors were observed. Another Australian cohort study (7) showed no increase in crude stroke incidence compared to previous Australian studies—Perth 191 in 1989, 157 in 1996, 120 in 2001; Melbourne 206 in 1999 (8). Stroke incidence may have population-specific biases. Changes were seen in stroke incidence and risk factors comparing the Oxford Community Stroke Project (1981–1984) and Oxford Vascular Study (2002–2004). Age- and gender-adjusted incidence of first ever stroke fell by 29%, there was a significant reduction in mortality rates for incident stroke and fatal or disabling stroke but no change in case-fatality rate, significant reductions in smoking, blood pressure, cholesterol, and significant increases in premorbid therapy with antiplatelets, lipid lowering agents, and antihypertensives. More recent data have also shown a decline in incidence TIA rates in a similar population, as well as 90-day risk of stroke following TIA, over a 10-year period.

**Table 3:** **Population-based incident community stroke studies**.

	Incidence rate	Cohort	Number of incident stroke events
Perth Community Stroke Study (1, 4–6)	Crude annual incidence first ever stroke 178/100,000	Population-based descriptive study	370
Perth Community Stroke Study (1, 4–6)	Incidence first ever stroke 104/100,000 (1989–1990)	Population-based descriptive study	251 (1989–1990)
	76/100,000 (1995–1996)		213 (1995–1996)
NEMESIS (8)	Crude annual incidence first ever stroke 206/100,000	Population-based case ascertainment descriptive study	276
Oxford Community Stroke Project (OCSP, 1981–1984)	OCSP—429/86,487	Population-based incidence studies	262 stroke
Oxford Vascular Study (OXVASC, 2002–2004) (9)	OXVASC—262/90,542		93 TIA
Third Stroke Registry in Tartu, Estonia (14)	188/100,000	Prospective population-based cohort study	451
Perth Community Stroke Study (1, 4–6)	Incidence first ever stroke 191/100,000 (1989–1990)	Population-based descriptive study	251 (1989–1990)
	157/100,000 (1995–1996)		213 (1995–1996)
	128/100,000 (2000–2001)		183 (2000–2001)

Of interest in our study was the lack of significant decline specifically in ischemic stroke for the period, but decline in ICH rates (for males). Some of the reasons for decline in incidence include the observation of decreasing risk rather than improving survival, improving primary and secondary prevention strategies and more stroke prevalence rather than incidence due to an increasing population.

Our study included analysis of a large cohort of patients (53,425) hospitalized with an incident primary stroke or TIA diagnosis over a 5-year period. As the data source is government mandated and routinely collected, as well as using standardized case definitions of ICD-10 coding, we believe our numbers reflect all admitted patients in the state, with minimal selection bias. Our rates may differ to other population stroke studies due to our large numbers, which may provide greater power to our analysis. These also include all public and private hospital admissions.

The limitations in using administrative data remain the absence of disease-specific data fields, lack of appropriate functional outcome measures, and potential for over-estimating length of stay or inaccurately diagnosing the reason for admission. This information is collected retrospectively and has a number of different uses. It is therefore important to understand that it can only provide a guide about trends and incidence. The data are collected for financial and planning purposes and have not been solely collected for research application.

We acknowledge limitations of our current study include the fact that the data analyzed is now at least 10 years old. We have seen an evolution in stroke data collection in the period since that time, with a stroke specific national clinical registry [Australian Stroke Clinical Registry—AuSCR (9)] being developed in Australia, to which a large number of hospitals nationally contribute data. There are plans to ultimately link Victorian Department of Health and Human Services administrative data with this hospital based registry, which will provide more a more detailed picture of the continuum of stroke patient hospital care. When compared with stroke registry data, inpatient administrative data have been observed to have a sensitivity of over 80% (10). It will be of interest to re-evaluate these trends using available more recent data also.

Our incidence definition is estimated, by using a 5-year clearance to ascertain first ever stroke or TIA (admitted). There is precedence for this approach, with evidence for prevalence pooling, using prevalence cases as first ever (incident) cases (11). Using clearance rates of 5 years, no more than 3% of cases would be estimated to be recurrent cases.

A similar study from the US used a large administrative database with approximately 20% case ascertainment of all hospital stroke discharges (377,544 in 1995) (12). Using ICD-9-CM primary and secondary stroke diagnostic codes to evaluate first ever and recurrent stroke incidence, they found the average age- and sex-adjusted incident rate for first ever stroke as 200 per 100,000. Corso et al. also found differences in stroke incidence from similar population studies around the world, postulating methodological and health-care system differences in each country (13, 14).

Our study captured only hospital admitted cases, hence does not represent a complete case ascertainment of all stroke and TIA cases in the defined population, as some cases may be managed by general practitioners or other specialists in the community. However, in the era of improved public education about stroke, it is common practice in Victoria and Australia generally for most patients with stroke or TIA to be sent to or attend hospital for urgent evaluation.

The power of this information lies in the large numbers available so that despite potential bias and error, useful assumptions can still be made. In future analyses, it will be interesting to assess the presence of ongoing decline in stroke rates, particularly with the advent of higher acuity acute stroke therapies and increased public education regarding stroke signs, as well as an aging population.

## Conclusion

There is emerging evidence of a decline in incident stroke rates in our population-based cohort. This may be due to the implementation of primary and secondary prevention strategies. In future, the use of stroke-specific registries will provide more definitive information regarding these trends and allow identification of associated factors, guiding future preventative efforts.

## Author Notes

First published as a conference abstract at the European Stroke Conference, Nice, France, 2014.

## Author Contributions

BC—conception and design, data acquisition and analysis, data interpretation, draft and critical revision, final approval, and accountability for work. VS—data acquisition, data analysis, data interpretation, draft and critical revision, final approval, and accountability for work. PC and JM—conception, design, draft and critical revision, final approval of work, and accountability for work.

## Conflict of Interest Statement

The authors declare that the research was conducted in the absence of any commercial or financial relationships that could be construed as a potential conflict of interest.
